# Beyond overlap: a Ro52-driven glandular-pulmonary phenotype at the interface of Sjögren disease and anti-synthetase syndrome in a multicentre cohort

**DOI:** 10.3389/fimmu.2026.1781334

**Published:** 2026-04-01

**Authors:** Gaetano La Rocca, Francesco Ferro, Giulia Morina, Andrea Pilato, Onorina Berardicurti, Giovanni Fulvio, Linda Carli, Carlo Vancheri, Gianluca Sambataro, Marta Mosca, Chiara Baldini

**Affiliations:** 1 Rheumatology Unit, University of Pisa, Department of Clinical and Experimental Medicine, Azienda Ospedaliero Universitaria Pisana, Pisa, Italy; 2 Regional Referral Center for Rare Lung Disease, University of Catania, Department of Clinical and Experimental Medicine, Policlinico “G. Rodolico-San Marco”, Catania, Italy; 3 Rheumatology and Clinical Immunology, University of Rome, Department of Medicine, School of Medicine, Rome, Italy; 4 Department of Medicine and Surgery, University of Enna “Kore”, Enna, Italy

**Keywords:** anti-Ro52 antibodies, anti-synthetase syndrome, interstitial lung disease, myositis-spectrum disorders, overlap syndromes, Sjögren’s disease

## Abstract

**Background:**

While Sjögren disease (SjD) overlap is known to modulate the clinical-serologic manifestations of other connective tissue diseases, the association with anti-synthetase syndrome (ASyS) has been seldom described in Caucasian cohorts. Anti-Ro52 autoantibodies, shared by both conditions, may blur early diagnostic attribution, particularly when glandular symptoms precede myositis-spectrum features.

**Objectives:**

To characterize the clinical phenotype, serological profile, and disease trajectory of patients fulfilling classification criteria for both SjD and ASyS.

**Methods:**

We conducted a multicentre retrospective study (2018-2025) across three Italian referral centres, including patients meeting both the 2016 ACR/EULAR SjD criteria and the Connor’s/provisional CLASS classification criteria for ASyS. Clinical features, serology, interstitial lung disease (ILD) characteristics, treatments, and outcomes were systematically collected. SjD activity was assessed using the ESSDAI and an adapted ESSDAI excluding the classic ASyS triad (pulmonary, articular and muscular domains).

**Results:**

Seventeen female patients of Caucasian ethnicity were identified. At the time of connective tissue disease diagnosis, 7 were classified as SjD and 10 received concomitant diagnoses; all reported early sicca symptoms. Regarding serology, anti-Ro52 antibodies were detected in all patients, most often (88%) in the absence of anti-Ro60 antibodies. Anti-synthetase antibodies were present in all cases, mainly non-Jo-1 specificities (77%), while anti-Jo-1 was observed in a minority of patients (23%). ILD was the leading organ involvement (14/17, 82%), typically with acute/subacute onset and NSIP or NSIP/OP patterns. ILD drove treatment decisions and accounted for four deaths, whereas most survivors showed stabilization or mild improvement under immunosuppressive treatment. Systemic assessment indicated a predominantly ASyS-driven phenotype: although ESSDAI was moderately elevated (median 15, IQR 7.5-21), the adapted ESSDAI markedly decreased (median 5, IQR 0-6.5), reflecting limited SjD-specific systemic activity.

**Conclusions:**

The SjD-ASyS overlap seem characterized by a coherent clinical-serological phenotype, defined by anti-Ro52-positive sicca presentation, non-Jo1 anti-ARS autoantibodies and early interstitial lung disease. Pulmonary involvement represents the main driver of disease course and outcomes in this subset. Recognition of this pattern may improve early identification of ASyS in Ro52-positive SjD presentations. Further prospective studies are warranted to clarify whether this subset reflects a distinct phenotype within the ASyS spectrum rather than a coincidental overlap of two independent diseases.

## 1 Introduction

Sjögren disease (SjD) is a systemic autoimmune disorder characterized by lymphocytic infiltration of exocrine glands, leading to sicca manifestations and the production of hallmark autoantibodies such as anti-Ro/SSA and anti-La/SSB (1).

Although traditionally viewed as a primarily glandular disease, SjD may coexist with other connective tissue disorders (CTDs), often shaping the clinical phenotype and serological signature of the overlapping condition (2). Conversely, autoantibodies typically associated with other CTDs may be detected in patients fulfilling SjD classification criteria even in the absence of overt clinical overlap, reflecting the immunological heterogeneity of the syndrome (3, 4).

Notably, the association between SjD and idiopathic inflammatory myopathies (IIMs), particularly anti-synthetase syndrome (ASyS), remains poorly explored (5, 6), with most available data on the ASyS-SjD overlap deriving from Asian cohorts (7) and limited evidence in Caucasian populations. ASyS is defined by the presence of anti-aminoacyl-tRNA synthetase (anti-ARS) autoantibodies and a characteristic constellation of manifestations including the triad of interstitial lung disease (ILD), myositis and arthritis (8). Anti-Ro52 antibodies have been consistently associated with a more severe ILD phenotype in CTD-associated ILD, characterized by poorer prognosis and, in some cases, a rapidly progressive course (9, 10). Although anti-Ro52 antibodies are frequently detected in SjD, where they may occur either in combination with anti-Ro60 or in isolation, their presence is also well documented in ASyS. Ro52 is an interferon-inducible E3 ubiquitin ligase with broad immunomodulatory functions, particularly on the IFN-driven inflammatory cascade (11).

This shared serological marker introduces diagnostic ambiguity and may obscure the recognition of underlying myositis-spectrum autoimmunity in patients initially labelled as SjD.

Recent work has begun to examine patients displaying features of both SjD and ASyS, yet most published studies approach the problem from the perspective of ASyS, identifying, within established myositis cohorts, individuals with positive anti-Ro or who also fulfill SjD criteria (7, 12). This framework does not entirely clarify the clinical trajectory of patients who present first with SjD-like manifestations (such as sicca symptoms or anti-Ro52 positivity) and only later develop features consistent with ASyS. Furthermore, the recently proposed provisional CLASS classification criteria for ASyS, which incorporate anti-Ro52 in the serological domain, underscore the need to revisit how early SjD-like presentations should be interpreted in the context of evolving inflammatory myopathy or ILD (13).

From this perspective, we conducted a multicentre observational study to characterize patients fulfilling both the 2016 ACR/EULAR SjD criteria and the Connor’s/CLASS criteria for ASyS.

The aim of this study was to delineate the clinical, serological and pulmonary phenotype of this overlap subset, to clarify its diagnostic trajectory, and to identify early features that may help clinicians distinguish SjD-associated manifestations from those signaling emerging ASyS. By doing so, we sought to inform a more refined diagnostic approach in patients presenting with SjD-like features who may in fact be evolving toward an anti-synthetase disease spectrum.

## 2 Materials and Methods

### 2.1 Study design and inclusion criteria

This was a multicentre, retrospective observational study including patients followed at the Rheumatology Units of Pisa University Hospital, Catania University Hospital (Policlinico G. Rodolico-San Marco), and Campus Bio-Medico University Hospital (Rome) between January 2018 and January 2025.

Patients were eligible for inclusion if they fulfilled both of the following:

1. 2016 ACR/EULAR classification criteria for SjD (14), based on anti-SSA positivity, objective glandular tests (Schirmer’s test and/or unstimulated salivary flow), and/or minor salivary gland biopsy (MSGB).
2. Connor’s classification criteria (15), as well as the provisional CLASS classification criteria for ASyS (13), applied retrospectively to all cases.

Patients were included regardless of whether SjD or ASyS was the first clinically assigned diagnosis, provided that available clinical and serological data allowed reconstruction of the initial presentation. Patients with unconfirmed or weakly positive anti-ARS reactivity on immunoblot were excluded.

The study was conducted according to the Declaration of Helsinki and approved by the Ethics Committee of Pisa University Hospital “Comitato Etico Regione Toscana - Area Vasta Nord Ovest (CEAVNO)” (protocol code: AIS-ILD; date of approval: 23/05/2024). All the patients signed their informed consent to participate to this study.

### 2.2 Serological assessment

All patients underwent ANA testing by indirect immunofluorescence, with titre and staining patterns recorded.

Anti-Ro/La antibodies were assessed by immunoblot, distinguishing anti-Ro52 from anti-Ro60.

Myositis-specific autoantibodies (MSA) were tested as part of routine clinical care, either at SjD diagnosis when overlap was suspected or during follow-up when clinical features suggestive of anti-synthetase syndrome emerged. MSA were assessed in participating centres using a standardized EUROIMMUN EUROLINE Autoimmune Inflammatory Myopathies (16 Ag) line blot panel, including Jo-1, PL-7, PL-12, EJ and OJ antigens. Semi-quantitative results (− to +++) were recorded. To minimize the chance of false-positives, anti-ARS positivity was considered reliable when confirmed on two independent samples and when at least one of the following criteria was met: (i) moderate-to-strong positivity (++/+++), (ii) a cytoplasmic ANA pattern on HEp-2 IIF, and/or (iii) clinical concordance, defined as at least two ASyS features among ILD, inflammatory arthritis/arthralgia, myositis, or characteristic cutaneous findings.

### 2.3 Clinical data collection

Information on xerostomia and xerophthalmia, Schirmer’s test results, and unstimulated salivary flow was retrieved from medical records.

Minor salivary gland biopsy (MSGB) was performed based on clinical indication and not mandated by protocol. Histopathology was graded using the Focus Score (16).

Electronic medical records were reviewed to collect the age at diagnosis of SjD, ASyS, and ILD.

ASyS classification followed Connor’s criteria (anti-ARS positivity plus ≥1 typical clinical feature: myositis, ILD, arthritis, persistent fever, Raynaud phenomenon, mechanic’s hands). The CLASS criteria score was calculated retrospectively to categorize patients as definite, probable, or possible ASyS.

SjD-related systemic involvement was assessed using the ESSDAI (17) at SjD diagnosis and follow-up. An adapted ESSDAI excluding articular, pulmonary, and muscular domains, representing the classic ASyS triad, was also calculated to distinguish SjD-driven activity from ASyS-driven manifestations.

Clinical features classically associated with ASyS but not included in ESSDAI (Raynaud phenomenon, mechanic’s hands, typical cutaneous rashes, dysphagia) were systematically recorded. Nailfold videocapillaroscopy (NVC) findings were assessed when available.

Therapeutic data, including immunosuppressive regimens and the organ involvement guiding treatment decisions, were collected.

### 2.4 Interstitial lung disease assessment

Respiratory history, including smoking exposure, chronic cough (defined as persistence >8 weeks) (18, 19), and dyspnea (grade ≥2 on the modified Medical Research Council scale) (20, 21), was recorded at ILD diagnosis and last follow-up.

ILD onset was categorized according to previously adopted definitions (22) as:

- acute/subacute: abrupt respiratory symptoms onset with progression within 1–3 months;
- chronic: insidious respiratory symptoms progressing over >3 months.

Rapidly Progressive (RP-ILD) was defined in the presence of acute or subacute respiratory deterioration with functional and/or radiologic progression occurring within a 3-month interval (23).

ILD diagnosis was confirmed in multidisciplinary discussion for all patients and high-resolution computed tomography (HRCT) patterns were classified by expert thoracic radiologists according to the 2013 ATS/ERS classification of the idiopathic interstitial pneumonias (24).

Pulmonary function tests (PFTs), including forced vital capacity (FVC% predicted) and diffusing capacity for carbon monoxide (DLCO% predicted), were recorded at ILD diagnosis and last follow-up, with percent predicted values derived from routine pulmonary function testing performed at each participating center.

### 2.5 Statistical analysis

Continuous variables are presented as mean ± SD or median (IQR), according to distribution. Categorical variables are presented as absolute numbers and percentages. Normality of continuous variables was assessed using the Shapiro–Wilk test and visual inspection of distributions.

Comparisons were performed using chi-square or Fisher’s exact test for categorical variables, and Student’s t-test or Mann-Whitney U test for continuous variables. Paired data were analysed using the Wilcoxon signed-rank test. Statistical significance was set at p < 0.05. For longitudinal analyses, baseline was defined according to the outcome of interest: for PFTs, baseline corresponded to the first available PFT at or after ILD diagnosis; for ESSDAI and adapted ESSDAI, baseline corresponded to the time of SjD or first CTD diagnosis.

Analyses were conducted using SPSS v.29.

## 3 Results

### 3.1 SjD and ASyS diagnosis and classification

Seventeen female patients of Caucasian ethnicity fulfilling both the 2016 ACR/EULAR SjD criteria and Connor’s/provisional CLASS classification criteria for ASyS were included. Particularly, 16/17 met the definition of definite ASyS of the provisional CLASS classification criteria with a median score of 7.25 (IQR 6.25-8), while one patient was defined as probable ASyS (score 5).

Median follow-up was 48 months (IQR 16-114). All patients reported xerostomia and/or xerophthalmia, and Schirmer’s test was positive in 13/17. Anti-Ro/SSA antibodies were detected in all individuals. Minor salivary gland biopsy was performed in seven patients, all of whom showed focal lymphocytic sialadenitis (Focus Score >1). In seven patients, sicca was the first clinical manifestation, leading to an initial diagnosis of SjD at a median age of 64 years (IQR 54-74). ASyS was subsequently diagnosed after a median interval of 3 years (IQR 3-8). The remaining ten patients were diagnosed with SjD and ASyS concomitantly (within six months), at a median age of 69 years (IQR 63.5-73).

### 3.2 Serology

ANA positivity was observed in 13/17 patients (76%), most commonly with a nuclear fine speckled pattern (8 cases, three of which with concurrent cytoplasmic staining). A pure cytoplasmic pattern was observed in three patients, and a homogeneous pattern in two (one with additional cytoplasmic staining).

Regarding anti-SSA specificities, isolated anti-Ro52 was the predominant profile (15/17; 88%). Two patients exhibited combined anti-Ro60/52 positivity, one of whom also had anti-La/SSB antibodies.

Anti-ARS autoantibodies were present in all patients. Non-Jo1 antibodies were the most frequently detected, namely anti-PL7 in 11 (65%), anti-PL12 in 1 (6%), anti-EJ in 1 (6%). Four patients had anti-Jo1 (23%).

Patient serological and demographic characteristics are summarized in **Table 1**.

**Table 1:** Demographic, clinical and serological characteristics of enrolled patients.

Features	Enrolled patients (n= 17)
Female, n (%)	17 (100)
Age at SjD diagnosis (years), median (IQR)	67 (53.5-72)
Age at ASyS diagnosis (years), median (IQR)	69 (58-73)
Follow-up (months), median (IQR)	48 (16-114)
First CTD diagnosis	
SjD, n (%)	7 (41)
Concomitant SjD/ASyS, n (%)	10 (59)
SjD classification criteria	
Anti-SSA, n (%)	17 (100)
double anti-Ro60/52, n (%)	2 (12)
isolated anti-Ro52, n (%)	15 (88)
anti-La, n (%)	2 (12)
Ocular tests positivity, n (%)	13 (76.5)
Positive MSGB (FS >1), n/N (%)	7/7 (100)
ASyS classification criteria	
Anti-ARS, n (%)	17 (100)
anti-Jo1, n (%)	4 (23)
anti-PL7, n (%)	11 (65)
anti-PL12, n (%)	1 (6)
anti-EJ, n (%)	1 (6)
CLASS criteria score, m (IQ)	7.25 (6.25-8)
Definite ASyS, n (%)	16 (94)
Probable ASyS, n (%)	1 (6)
Treatment	
Systemic glucocorticoids, n (%)	17 (100)
Hydroxychloroquine, n (%)	3 (18)
First-line DMARDs	
MMF, n (%)	9 (53)
AZA, n (%)	4 (23)
CYC, n (%)	3 (18)
CyA, n (%)	1 (6)
Rituximab treatment, n (%)	3 (18)
Nintedanib add-on treatment, n (%)	1 (6)

SjD, Sjögren’s disease; IQR, interquartile range; ASyS, antisynthetase syndrome; CTD, connective tissue disease; MSGB, minor salivary gland biopsy; FS, focus score; DMARDs, disease-modifying antirheumatic drugs; MMF, mycophenolate mofetil; AZA, azathioprine; CYC, cyclophosphamide; CyA, cyclosporine A.

### 3.3 SjD- and ASyS-related clinical manifestations

At the time of the first CTD diagnosis, oral or ocular sicca symptoms were present in 12 (70%) and 14 (82%) patients, respectively; all patients developed both by the end of follow-up.

Fourteen patients (82%) exhibited at least one manifestation of the classic ASyS triad at disease onset. Isolated ILD was most common (n=8; 47%), followed by ILD with arthritis (n=3; 18%) and ILD with myositis (n=2; 12%). Only one (6%) patient presented the full triad at onset.

During follow-up, the pattern of major organ involvement remained stable, with only two patients developing *de novo* ASyS-related manifestations.

Typical B-cell driven SjD extraglandular features were rare. Lymphadenopathy (n=4) and hypergammaglobulinemic purpura (n=3) were the most frequent. Laboratory abnormalities included hypergammaglobulinemia in five patients and a monoclonal component in three. RF positivity was recorded in two cases and hypocomplementemia (low C3) in one patient.

Median ESSDAI at SjD diagnosis was 15 (IQR 7.5-21). The adapted ESSDAI, excluding domains overlapping with the typical ASyS triad (muscular, articular, pulmonary), decreased the median score to 5 (IQR 0-6.5), highlighting the contribution of ASyS-related involvement to apparent SjD activity. Clinical and laboratory features at onset and last follow-up are reported in **Tables 2**, **3**.

**Table 2:** ASyS and SjD-related clinical manifestations at the time of the first CTD diagnosis and by the last follow-up.

Clinical manifestations	Disease onset	Last follow-up
ASyS triad manifestations		
Isolated ILD, n (%)	8 (47)	9 (52)
ILD and arthritis, n (%)	3 (18)	3 (18)
ILD and myositis, n (%)	2 (12)	3 (18)
ILD, myositis and arthritis, n (%)	1 (6)	2 (12)
Isolated myositis, n (%)	0 (0)	0 (0)
Isolated arthritis, n (%)	0 (0)	0 (0)
ASyS- and SjD -associated clinical manifestations		
Inflammatory arthralgia, n (%)	12 (71)	13 (77)
Persistent Fever, n (%)	3 (18)	3 (18)
Raynaud’s phenomenon, n (%)	6 (36)	6 (35)
NVC SSc/PDM pattern, n/N (%)	4/16 (25)	6/16 (37)
Mechanic’s hands, n (%)	2 (12)	6 (35)
Typical skin rash, n (%)	4 (23)	5 (29)
Dysphagia, n (%)	2 (12)	3 (18)
Xerostomia, n (%)	12 (71)	17 (100)
Xerophthalmia, n (%)	14 (82)	17 (100)
SGE, n (%)	0 (0)	0 (0)
Lymphadenopathies, n (%)	4 (24)	4 (24)
Purpura, n (%)	3 (18)	3 (18)
Renal involvement, n (%)	0 (0)	0 (0)
Hematologic involvement, n (%)	0 (0)	1 (6)
PNS involvement, n (%)	0 (0)	0 (0)
CNS involvement, n (%)	1 (6)	1 (6)

NVC, nailfold videocapillaroscopy; SSc/PDM pattern, scleroderma/polymyositis–dermatomyositis pattern; SGE, Salivary Glands Enlargement; PNS, Peripheral Nervous System; CNS, Central Nervous System.

**Table 3:** ASyS and SjD-related laboratory features at the time of the first CTD diagnosis and by the last follow-up.

Laboratory manifestations	Disease onset	Last follow-up
Hypergammaglobulinemia, n (%)	5 (29)	3 (18)
Monoclonal component, n (%)	3 (18)	3 (18)
RF positivity, n (%)	2 (12)	2 (12)
Cryoglobulinemia, n (%)	0 (0)	0 (0)
Low C3/C4, n (%)	0 (0) / 0 (0)	1 (6) / 0 (0)
Raised CK, n (%)	3 (18)	1 (6)
Raised CRP, n (%)	6 (36)	8 (47)

RF, rheumatoid factor; CK, creatine kinase; CRP, C-reactive protein; Hypergammaglobulinemia, raised CK and raised CRP were defined according to the upper limit of normal of the local laboratory reference ranges at each participating center.

### 3.4 ILD characteristics

ILD was the predominant organ involvement, already present in 14/17 patients at the time of the first CTD diagnosis and developing later in 3 patients after a median interval of 84 months (IQR 48-252). Median age at ILD diagnosis was 66 years (IQR 56.7-71). ILD presentation was acute/subacute in 13 patients (76%), including three cases fulfilling criteria for RP-ILD, while four patients showed a chronic onset. Only one patient was asymptomatic at ILD detection.

During follow-up, chronic dyspnoea and cough developed in 15 (88%) and 9 (53%) patients, respectively.

On HRCT, non-specific interstitial pneumonia (NSIP) was the most frequent pattern (65%), followed by NSIP with organizing pneumonia overlap (22%). One patient showed a pure organizing pneumonia pattern and one a usual interstitial pneumonia pattern. Fibrotic features on HRCT were observed in 13 patients (76%).

PFT (available for 15 patients) at ILD diagnosis showed a median FVC of 76.15% (IQR 61-94) and median DLCO of 57.5% (IQR 43.5-70.5). Two patients were unable to perform baseline pulmonary function testing because of severe desaturation.

### 3.5 Treatment

All patients received systemic glucocorticoids in combination with at least one immunosuppressive agent. In 15 patients (88%), ILD was the main determinant guiding treatment decisions; in the remaining two, active myositis initially guided therapy, although ILD subsequently became the leading target. First-line immunosuppressive therapies included mycophenolate mofetil (n=9), azathioprine (n=4), cyclophosphamide (n=3), and cyclosporine A (n=1). Hydroxychloroquine was prescribed in three patients and IVIG in one. Treatment escalation was required in five cases due to ILD progression: four patients initially treated with azathioprine were switched to mycophenolate (n=2) or cyclophosphamide (n=2), and rituximab was added in one patient already receiving mycophenolate. In addition, two other patients received rituximab during the disease course as maintenance therapy after first-line treatment with cyclosporine A and cyclophosphamide, respectively. Finally, one patient received nintedanib for progressive pulmonary fibrosis.

### 3.6 Disease course

At last follow-up, seven patients required long-term oxygen therapy, and four had died, all from ILD. In all four cases, ILD was present at the time of CTD diagnosis.

**Figure 1:**
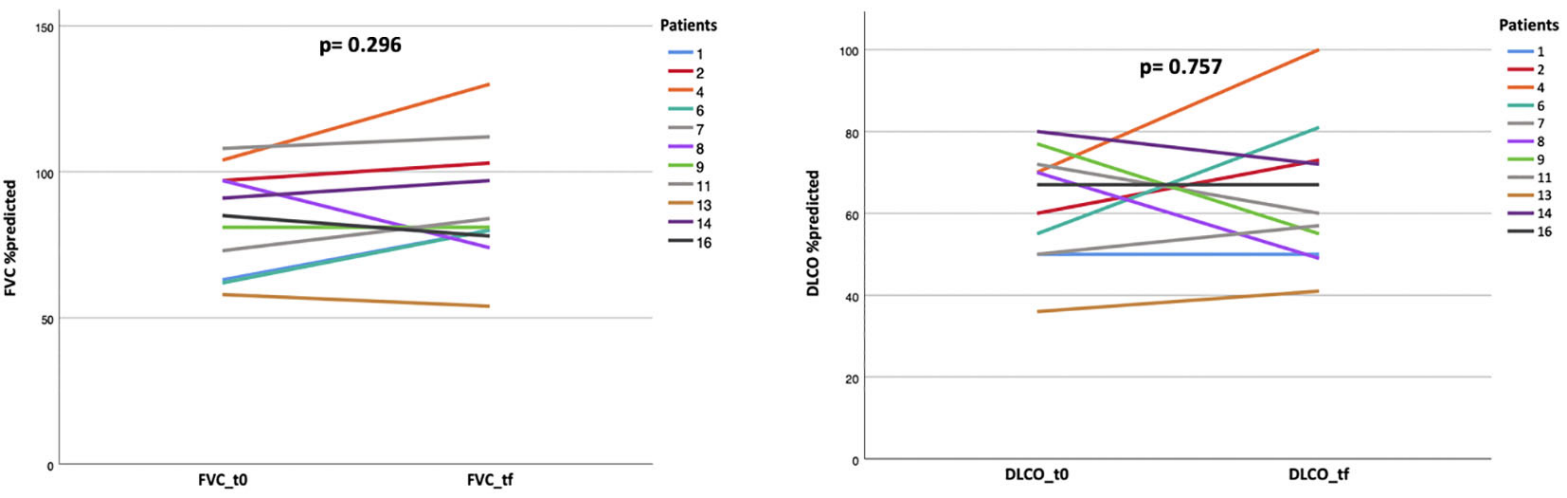
Individual trajectories of pulmonary function from ILD diagnosis to the last follow-up. Among patients with paired PFT data (n=11), forced vital capacity (FVC%, left) showed overall stability with a mild upward trend, although three patients (IDs 8, 9, and 13) experienced decline. Similarly, diffusing capacity for carbon monoxide (DLCO%, right) remained stable or improved in most cases, except for three patients (IDs 8, 9, and 14). Group-level changes in FVC and DLCO did not reach statistical significance. Patient identifiers (pt 1-17) are consistent across **Figures 1**, **2**.

Among patients with paired follow-up data (n=11), PFT showed no significant deterioration: median FVC increased from 76.15% to 81% predicted (p=0.296), while DLCO remained stable (57.5% to 60%, p=0.757). Of note, however, three patients with available baseline PFT were unable to repeat the test due to severe desaturation during progression. PFT trajectories are shown in **Figure 1**.

**Figure 2:**
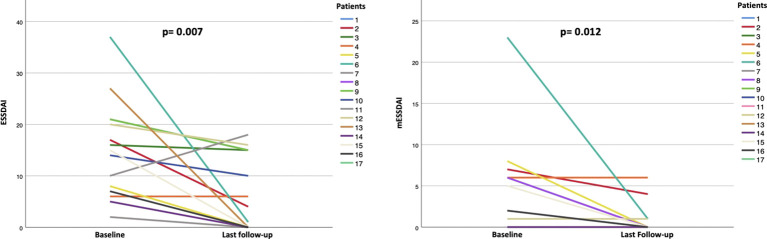
Individual changes in ESSDAI (left graph) and adapted ESSDAI (right graph) from SjD diagnosis to the last follow-up. Total ESSDAI decreased in almost all patients from SjD diagnosis to last follow-up; only patient 4 showed stable values and patient 7 experienced an increase over time. Overall, median total ESSDAI significantly declined (p = 0.007). The adapted ESSDAI, excluding pulmonary, articular, and muscular domains, remained stable or decreased in all patients except patient 4, who showed persistent disease activity in typical “SjD-driven domains”. Median adapted ESSDAI significantly declined over time (p = 0.012).

Overall disease activity decreased significantly. ESSDAI was available for all patients (n=17) and declined from 15 (IQR 7.5-21) to 4 (IQR 0-15) (p=0.007), while the adapted ESSDAI decreased from 5 (IQR 0-6.5) to 0 (IQR 0-1) (p=0.012). Changes over time are illustrated in **Figure 2**.

## 4 Discussion

In this multicentre study we characterized a series of patients fulfilling both the 2016 ACR/EULAR classification criteria for Sjögren’s disease (SjD) and the Connor’s/provisional CLASS classification criteria for anti-synthetase syndrome (ASyS), an overlap scenario that remains insufficiently addressed in the literature. To our knowledge, this represents the largest European case series systematically evaluated to date, supported by a prolonged follow-up, which provides a robust framework for examining the clinical-serological features and trajectory of this uncommon phenotype.

Our cohort included 17 females, all of whom exhibited sicca symptoms and anti-SSA positivity, with focal lymphocytic sialadenitis confirmed in the subset undergoing biopsy. Isolated anti-Ro52 was the predominant serological profile, accompanied by anti-ARS antibodies in every case, most frequently non-Jo1 specificities. ILD represented the main systemic manifestation, typically showing NSIP or NSIP-OP patterns, and was the principal driver of immunosuppressive treatment. During follow-up, four patients died from ILD progression, whereas most others experienced stable or improved pulmonary function and a decline in overall disease activity measured by ESSDAI.

Although sicca symptoms and anti-Ro/SSA antibodies have been described in ASyS, the recognition of true SjD-ASyS coexistence has been limited, and the early clinical interplay between the two conditions remains insufficiently characterized (12, 25). Our findings provide a complementary perspective by capturing individuals as they were initially recognized in routine clinical practice, often within a SjD-oriented diagnostic pathway, thereby offering insight into the early expression and subsequent evolution of this overlap phenotype.

Indeed, a notable and reproducible observation across our series is that SjD frequently represents the first assigned diagnosis. Seven patients were initially classified as primary SjD, while the other ten received both SjD and ASyS diagnoses within a short interval, all displaying symptoms of sicca and either objective ocular or salivary gland abnormalities early in their disease course. This pattern mirrors what has been reported in a recent Chinese study by Qiu et al., in which 42 patients with ASyS-SjD carried a previous diagnosis of SjD (7). Similarly, a recent case-based literature review confirms that in most published ASyS-SjD reports, SjD is diagnosed first or concurrently, whereas ASyS-specific features typically emerge later (26).

The consistency of this finding across ethnically different cohorts suggests that a SjD-first presentation is unlikely to be incidental and may represent a recurrent clinical pattern within this overlap, in which glandular and pulmonary involvement could reflect shared immune mechanisms.

The serological profile of our patients provides additional context for this hypothesis. Anti-Ro52 antibodies were detected in all individuals, most often in isolation. The presence of anti-Ro52 appears to play a central role in anchoring early diagnostic reasoning toward SjD when combined with glandular symptoms. Yet, anti-Ro52 antibodies have equally strong associations with type-I-interferon activation, ILD severity and myositis-spectrum autoimmunity (27, 28). Therefore, these patients could represent a bridging clinical phenotype associated with an antibody specificity shared between SjD and ASyS, rather than a purely coincidental overlap of two primary conditions.

Pulmonary involvement represented the predominant systemic manifestation in this phenotype and was present at the time of CTD diagnosis in most patients. NSIP and NSIP/OP, the main HRCT patterns observed, are not specific and are frequently reported both in ASyS- and SjD-associated ILD. However, the overall context in our cohort, including the frequent acute/subacute presentation and ILD-driven outcomes, was more closely aligned with an ASyS-spectrum ILD than with classical SjD lung involvement (28, 29). The aggressive behavior of ILD in a subset of individuals, including cases of RP-ILD, underscores the importance of early recognition of this overlap phenotype, particularly in patients presenting initially with sicca symptoms and isolated Ro52 positivity.

Extra-glandular features traditionally associated with SjD were infrequent, and laboratory markers of B-cell hyperactivity, such as hypergammaglobulinemia, rheumatoid factor positivity or hypocomplementemia, were uncommon. Comparison between ESSDAI and adapted ESSDAI values suggested that most measurable systemic burden stemmed from typical ASyS-related domains, rather than from SjD B-driven manifestations. Taken together, these findings suggest that while glandular involvement fulfils SjD classification criteria and defines the early clinical presentation, the dominant systemic trajectory in these patients is more consistent with ASyS.

Our clinical data align with transcriptomic findings of Qiu et al. identifying inflammatory, extracellular-matrix and neutrophil-associated signatures in ASyS-SjD ILD, which may parallel early immune events at epithelial barrier tissues (7). Moreover, findings from recent mechanistic studies reporting anti-ARS and anti-Ro52 antibodies in bronchoalveolar lavage fluid and salivary gland tissue provide biological plausibility for a model in which glandular and pulmonary involvement may be linked by shared mucosal immune activation (30). While our clinical data cannot directly test this hypothesis, the reproducible SjD-first recognition pattern, the Ro52-dominant serology and the tendency toward ILD-driven systemic evolution are consistent with such an integrated perspective.

Furthermore, taken together these observations have important clinical implications. Ro52-positive patients presenting with sicca symptoms should be comprehensively evaluated and reassessed if they develop ILD, arthritis or even subtle systemic inflammation, as these features may indicate evolving ASyS. Timely recognition of this overlap phenotype may improve organ-specific outcomes, particularly ILD, which remains the major determinant of morbidity and mortality. Our findings also prompt reconsideration of how anti-SSA positivity is interpreted within current SjD classification frameworks. Because the 2016 ACR/EULAR criteria treat Ro52 and Ro60 as a single serologic entity, they may inadvertently capture Ro52-positive sicca presentations whose clinical evolution aligns more with an ASyS-related phenotype than with classical SjD. This does not undermine the criteria, but highlights their ability to encompass phenotypes at the interface of SjD and myositis, with Ro52 acting as a shared immunologic denominator.

A more flexible, bidirectional diagnostic approach may therefore be required, one that recognizes the extent to which Ro52-associated immunity can blur traditional boundaries between SjD and ASyS.

Our study has limitations, mostly related to its retrospective design and inherent to the rarity of this overlap. Given the small sample size and the exploratory nature of the study, statistical analyses were primarily descriptive and intended to support estimation; accordingly, p values should be interpreted cautiously and as descriptive rather than confirmatory. The limited availability of salivary gland biopsies also restricts the ability to derive mechanistic conclusions. PFT were reported using %predicted values, in line with clinical practice at the time of data collection; however, more recent ATS/ERS recommendations favor interpretation based on z-scores and lower limits of normal. In addition, longitudinal PFT analyses are limited by informative missingness, as patients with more severe disease progression were less likely to have paired follow-up measurements. Finally, non-centralized HRCT interpretation and serological testing may have introduced a degree of inter-center variability.

On the other hand, the strengths of this study include its multicentre design, the detailed reconstruction of diagnostic trajectories and clinical outcomes, including disease activity in each ESSDAI domain. Of note, the recently proposed provisional CLASS classification criteria were applied retrospectively in this cohort. To our knowledge, this study is among the first reports employing the CLASS criteria for classification of ASyS patients in a real-world setting.

Our findings describe a reproducible clinical and serological pattern observed across three Italian referral centres, in which ILD represents the leading manifestation, accompanied by a characteristic autoantibody profile frequently marked by isolated anti-Ro52 positivity and non-Jo1 anti-ARS antibodies. This combination is unlikely to reflect the simple coexistence of SjD and ASyS. Rather, it points to a coherent clinical phenotype in which early glandular and pulmonary involvement may occur in parallel, perhaps reflecting a mucosal site of autoimmune activation. Whether this phenotype represents a specific subset within the ASyS or a distinct clinical entity requires further investigation. Prospective studies integrating longitudinal assessment, refined serological profiling and salivary-gland tissue analysis will be crucial to delineate its position within CTD nosology.

## Data Availability

The raw data supporting the conclusions of this article will be made available by the authors, without undue reservation.
